# Fluorescent advanced glycation end products in type 2 diabetes and its association with diabetes duration, hemoglobin A1c, and diabetic complications

**DOI:** 10.3389/fnut.2022.1083872

**Published:** 2022-12-15

**Authors:** Rui Liu, Mengyao Zhang, Li Xu, Jingjin Liu, Pingan Yang, Min Li, Jie Qin

**Affiliations:** ^1^Department of Endocrinology, Shanxi Provincial People’s Hospital, Fifth Hospital of Shanxi Medical University, Taiyuan, China; ^2^Department of Cardiology, Shanxi Provincial People’s Hospital, Fifth Hospital of Shanxi Medical University, Taiyuan, China

**Keywords:** fluorescent advanced glycation end products (fAGEs), type 2 diabetes mellitus, glycosylation, carotid intima media thickness (CIMT), diabetic complications

## Abstract

**Background:**

Fluorescent advanced glycation end products (fAGEs) are generated through the Maillard reaction between reducing sugars and amino compounds. fAGEs accumulation in human bodies have been confirmed to be related to many chronic diseases. To date, the correlations between serum fAGEs levels and clinical parameters or carotid intima media thickness (CIMT) in patients with T2DM remain unclear. Thus, this study aimed to investigate the relationship between serum AGEs levels and clinical parameters or CIMT in patients with T2DM.

**Method:**

A total of 131 patients with diabetes and 30 healthy controls were enrolled. Patients were divided into three groups according to diabetes duration, including ≤5, 5–10, and ≥10 years. Serum fAGEs, protein oxidation products, clinical parameters, and CIMT were determined.

**Results:**

The result showed that levels of fAGEs and protein oxidation products increased with the increasing duration of diabetics. Pearson correlation coefficients of fAGEs versus hemoglobin A1c (HbA1c) were >0.5 in patients with diabetes duration ≥10 years. A continued increase in fAGEs might cause the increase of HbA1c, urinary albumin/creatinine ratio (UACR) and CIMT in patients with T2DM.

**Conclusion:**

Our study suggested that levels of fAGEs could be considered as an indicator for duration of diabetics and carotid atherosclerosis. Diabetes duration and smoking might have a synergistic effect on the increment of fAGEs levels, as evidence by the results of correlation analysis in patients with long-duration diabetics (≥10 years) and smoking. The determination of fAGEs might be helpful to advance our knowledge on the overall risk of complications in patients with T2DM.

## 1 Introduction

Advanced glycation end products (AGEs) are generated through the Maillard reaction between reducing sugars (such as glucose and fructose) and amino compounds (1, 2). This reaction occurs both in heat processed foods (3) or *in vivo* (4). It has been reported that dietary AGEs might be released into the blood stream or directly gain entry into the systemic circulation (5). Accumulation of dietary AGEs in blood stream have been confirmed to be related to many chronic diseases, such as kidney disease (6), diabetes (7), atherosclerosis (8), Alzheimer’s disease (AD) (9) or tumor (10). Therefore, AGEs have received much attention not only in food science but also in clinical research.

**FIGURE 1:**
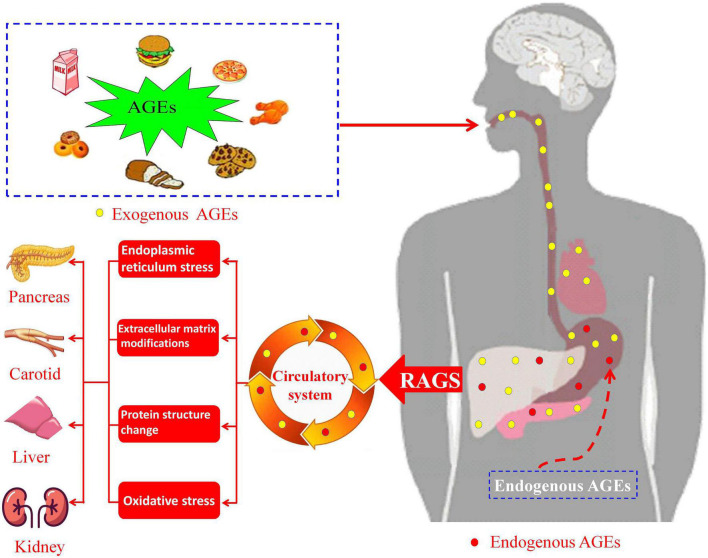
Schematic pathway of advanced glycation end products (AGEs) intake and formation in human body and AGEs-induced diseases.

Usually, AGEs in the body are mainly obtained primarily through dietary intake (exogenous AGEs) or self-metabolism (endogenous AGEs). Exogenous AGEs were generated in foods high in fat and protein content (11, 12), and endogenous AGEs are formed in body due to altered glucose metabolism (13, 14). These compounds would eventually enter the blood circulatory system through digestion and absorption (Figure 1). Therefore, a high-AGEs diet or higher levels of endogenous AGEs would induce the accumulation of AGEs in human tissues, resulting in the organ injury and dysfunction (such as pancreas, carotid, liver, and kidney). At present, relationships between AGEs and human disease have been previously discussed. Koska et al. (15) investigated the relationship between AGEs and incident cardiovascular disease (CVD) in patients with type 2 diabetes mellitus (T2DM), which showed that higher levels of AGEs are associated with increased incident CVD. Akram et al. (16) investigated that AGEs levels in gingival crevicular fluid of chronic periodontitis, which indicated that AGEs contents are higher in patients with T2DM. Cai et al. (17) also suggested that binding of AGEs and AGEs receptors could induce oxidative stress, leading to islet cell dysfunction and insulin resistance (18). Uribarri et al. (19) investigated that the relationship between dietary intake of AGEs and insulin resistance, which indicated that exogenous AGEs might contribute to insulin resistance in patients with T2DM. Diet-derived AGEs might be released into the systemic circulation, which might participate in the progress of diabetes and uremia (20). In addition to endogenous AGEs and dietary AGEs, cigarette smoke is also one source of AGEs and the main induction factors for AGEs formation (21, 22). These studies mainly focused on the pathophysiological effect of AGEs in diseases and influencing factors of AGEs generation *in vivo*. However, the correlations between serum AGEs levels and clinical parameters, carotid intima media thickness (CIMT), or smoking in patients with T2DM remain unclear.

**FIGURE 2:**
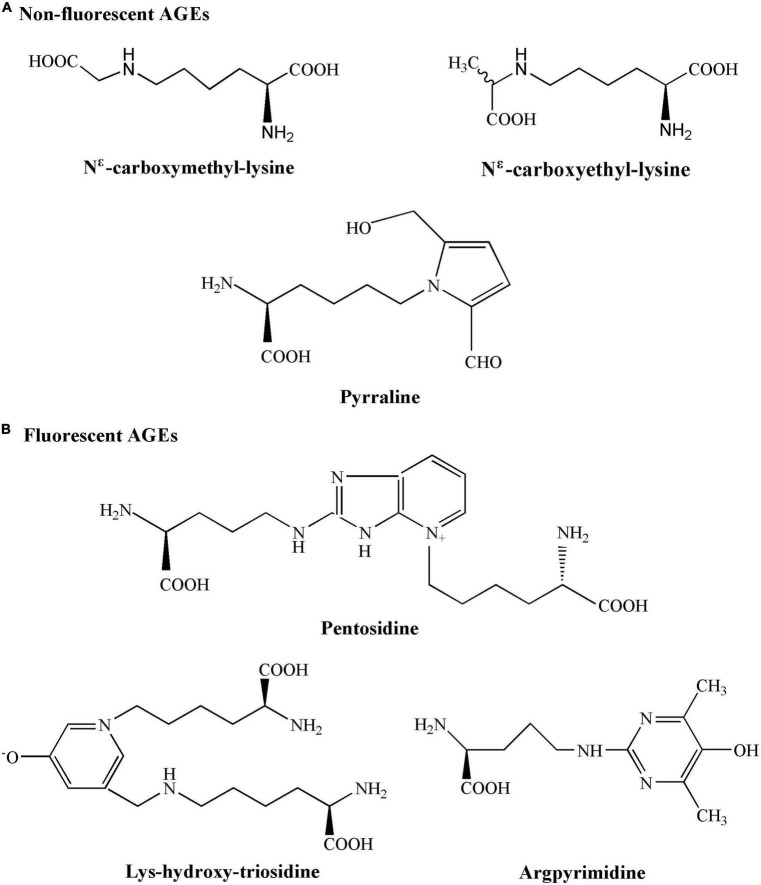
Chemical structures of non-fluorescent advanced glycation end products (AGEs) and fluorescent AGEs.

According to different spectral fluorescence properties, AGEs could be divided into non-fluorescent AGEs (N^ε^ -carboxymethyl-lysine, N^ε^ -carboxyethyl-lysine, and pyrraline) and fluorescent AGEs (fAGEs) (Figure 2; 23, 24). Many AGEs are capable of forming cross-links between proteins and most of them have fluorescent properties (such as pentosidine, lys-hydroxy-triosidine, and argpyrimidine). The fluorescence intensity was then used to measure the fAGEs concentrations in serum due to the autofluorescence characteristics of fAGEs (25). Serum fAGEs levels could be used as a reference for long-term blood glucose control in diabetes (26). Therefore, the objective of this work was to evaluate the correlation between serum fAGEs levels and CIMT, smoking or clinical parameters, such as hemoglobin A1c (HbA1c), serum uric acid (UA), triglyceride (TG), low-density lipoprotein (LDL), high-density lipoprotein (HDL), cholesterol (CHO), urinary albumin/creatinine ratio (UACR), in patients with T2DM, thereby providing some valuable references and guidelines for understanding the development and progression of diabetic complications.

## 2 Materials and methods

### 2.1 Subjects

According to the diagnostic criteria of WHO1999, all the subjects were 131 patients with type 2 diabetes hospitalized in Shanxi People’s Hospital from June 2021 to December 2021. A total of 30 healthy subjects who for health check-up were enrolled as the control group. These patients were divided into four groups based on the different duration of diabetics: (1) control (*n* = 30), (2) ≤5 years (*n* = 49), (3) 5–10 years (*n* = 33), and (4) ≥10 years (*n* = 49). Besides, these patients were also classified into two groups according to carotid intima media thickness (CIMT ≥ 1 mm or CIMT < 1 mm) and smoking (smoking or no smoking), respectively.

### 2.2 Ethical guidelines

The present study was approved by the Institutional Ethics Committee of Shanxi Provincial People’s Hospital, Taiyuan, Shanxi Province, China (approval no. 2022023), and was conducted in accordance with the Helsinki Declaration. The main purpose and method of the study were explained to all participants. All participants signed the informed consent form, and they could withdraw from the study, at any time.

### 2.3 Inclusion and exclusion criteria

The inclusion criteria in this study were as follows: (1) signing the written informed consent form, (2) T2DM, (3) BMI: 19–35 kg/m^2^. Exclusion criteria: (1) other types of diabetes; (2) ketosis or ketoacidosis, hyperosmolar coma, and severe stress within half a year, (3) family history of mental illness or alcohol users, (4) individuals that had used antibiotics, probiotics, non-steroidal anti-inflammatory drugs, and/or steroids within the past 3 months, (5) lactating or pregnant females, (6) patients with other serious illnesses.

### 2.4 Patients and blood sample collections

This study was designed to assess the correlation between serum fAGEs and AGEs-related complications in patients with T2DM, thereby predicting the severity of diabetic complications by fAGEs levels. Briefly, 131 T2DM patients and 30 healthy subjects (75 males and 86 females) in the department of endocrinology from Shanxi Provincial People’s Hospital were selected. All blood tests were determined after an overnight fast of >8 h.

### 2.5 Measurements

Hemoglobin A1c levels of diabetics patients were determined by the method reported by Thevarajah et al. (27) with slight modification. Briefly, the centrifuged blood samples were analyzed by a trained and calibrated investigator using ion-exchange high-performance liquid chromatography (ARKRAY Inc. Kyoto, Japan). Individuals with HbA1c levels of < 6.0 and ≥6.0% were considered normoglycemic and hyperglycemic, respectively. Blood and urine tests were performed at the clinical laboratory. That is to say, blood and urine sample were tested at a certified central laboratory for levels of UA, TG, CHO, HDL, LDL, and UACR according to standard procedures.

### 2.6 CIMT

Carotid intima media thickness were scanned by the method reported by Jun et al. (28). The subjects were supine, and the neck was fully exposed. Meanwhile, the head of subjects turned to the side away from the ultrasound physician. CIMT measurement was taken using LOGIQ 7 machine equipped with a 10 MHz linear transducer (GE, Healthcare, Milwaukee, WI, USA). CIMT value was scanned at three points: the far wall of the mid and the distal common carotid artery, and 1.0 cm proximal to the carotid bulb. The mean value of the three measurements on each side was used as the CIMT value (29). Usually, focal wall thickening >50% of the surrounding CIMT, or its CIMT of 1.5 mm was identified as carotid plaque (30).

### 2.7 Fluorescence intensity of AGEs

The fluorescence intensity of AGEs in serum samples was determined using the method described by Ferrer et al. (31) with some modifications. Briefly, 5 ml fasting blood was collected, and centrifuged at 1000 g for 10 min to separate the serum. FAGEs levels were evaluated on a fluorescence spectrometer (PerkinElmer LS-55). The excitation and emission wavelength was 370 and 440 nm, respectively. The slit width was 5.0 nm. The fluorescence intensities of AGEs were measured against reagent blank prepared with the same reagent concentrations.

### 2.8 Determination of protein oxidation products

In general, POPs include dityrosine, N’-formylkynurenine, and kynurenine (25). These compounds were quantified by the method reported by Ou et al. (25) with modifications. Based on different fluorescence intensities of POPs, the excitation wavelengths were chosen at 330, 325, and 365 nm, respectively; the emission wavelengths were recorded at 415, 434, and 480 nm, respectively, for the quantification of dityrosine, N’-formylkynurenine, and kynurenine, respectively. Then, 5 ml fasting blood was collected, and centrifuged at 1000 g for 10 min to separate the serum.

The fluorescence intensities of POPs were measured against reagent blank prepared with the same reagent concentrations. Besides, a fluorescence spectrometer (PerkinElmer LS-55) was used to quantify the POPs contents.

### 2.9 Statistics

All statistical analyses were performed using the statistix version 9.0 software (Analytical Software, Tallahassee, FL, USA) and GraphPad Prism 8.0 software (GraphPad, San Jose, CA, USA). Continuous data with a normal distribution were expressed as mean value ± SE, whereas non-normal distributed data are expressed as medians (quartile). The statistical significance (*P* < 0.05) was evaluated using unpaired Student’s *t*-test and Pearson’s correlation coefficient r.

## 3 Results

### 3.1 Baseline characteristics

Baseline characteristics were showed in Table 1. A total of 161 individuals were enrolled in this study: 131 patients with type 2 diabetes and 30 healthy subjects, including 75 males and 86 females. According to the duration of diabetics, 131 patients with T2DM were divided into three groups: ≤5 years (*n* = 49), 5–10 years (*n* = 33), and ≥10 years (*n* = 49). The baseline characteristics showed that there was no significant difference in age, sex and BMI among the groups. There were significant differences in HbA1c and UACR (*P* < 0.05), but no significant differences were found in UA, TG, LDL, HDL, and CHO (Table 1).

**TABLE 1:** Clinical and biochemical characteristics of participants.

		Diabetes duration			
Characteristic	Control subjects	≤5	5–10	≥10	*P*-value
Subjects (*n*)	30	49	33	49	NA**
Age (years)	41 ± 13	51 ± 17	59 ± 13	64 ± 15	0.3063
Sex (male/female)	12/18	28/21	13/20	22/27	0.1332
BMI (kg/m^2^)	24.3 ± 6.3	32.4 ± 2.4	30.8 ± 3.7	27.1 ± 4.4	0.3454
HBA1c	5.6 ± 0.6	5.9 ± 2.1	6.7 ± 1.6	9.2 ± 1.7	0.0484*
UACR	4.0 ± 1.3	9.9 ± 2.7	11.1 ± 2.4	18.4 ± 3.7	0.0013*
UA	306 ± 44	329 ± 59	336 ± 48	439 ± 53	0.0526
TG	2.8 ± 1.1	2.4 ± 0.5	2.0 ± 1.2	1.6 ± 0.8	0.4806
LDL	2.7 ± 0.6	2.8 ± 0.7	3.0 ± 1.2	2.7 ± 0.8	0.9594
HDL	1.2 ± 0.3	1.0 ± 0.1	1.1 ± 0.4	1.1 ± 0.3	0.8740
CHO	4.3 ± 1.4	4.4 ± 1.0	4.6 ± 1.1	4.3 ± 0.6	0.9828

*P-values reflect differences between means of control subjects and diabetes at baseline (*P* < 0.05).

**NA, not applicable.

### 3.2 POPs and fAGEs

As shown in Table 2, levels of dityrosine, N’-formylkynurenine, and kynurenine in patients with long-duration diabetics (≥10 years) were significantly higher than that in patients with short-duration diabetics (≤5 and 5–10 years), which meant that duration of diabetics could significantly affect protein oxidation. Furthermore, a similar trend was observed in the amounts of fAGEs. In patients with long-duration diabetics (≥10 years), serum fluorescence intensity of AGEs (34.7 ± 1.2) were significantly (*P* < 0.05) higher than that in patients with short-duration diabetics (5–10 and ≤5 years) (24.7 ± 1.5 and 19.7 ± 1.2, respectively).

**TABLE 2:** Fluorescence intensities of advanced glycation end products (AGEs) and protein oxidation products (POPs) in healthy subjects and patients with type 2 diabetes mellitus (T2DM)*.

		Diabetes duration		
Fluorescence intensity	Healthy subjects (*n* = 30)	≤5 (*n* = 49)	5–10 (*n* = 33)	≥10 (*n* = 49)
fAGEs*	17.7 ± 1.6^c^	19.7 ± 1.2^c^	24.7 ± 1.5^b^	34.7 ± 1.2^a^
Dityrosine*	19.7 ± 2.0^b^	19.1 ± 1.6^b^	22.6 ± 1.9^b^	33.2 ± 1.6^a^
N’-formylkynurenine*	24.6 ± 2.2^b^	20.9 ± 1.7^b^	26.0 ± 2.1^b^	36.8 ± 1.7^a^
Kynurenine*	23.6 ± 1.8^d^	29.4 ± 1.4^c^	34.8 ± 1.8^b^	43.7 ± 1.4^a^

*Different letters (a–d) in the same row indicate significant differences (*P* < 0.05).

### 3.3 Correlation between fAGEs and clinical parameters in patients with T2DM

**FIGURE 3:**
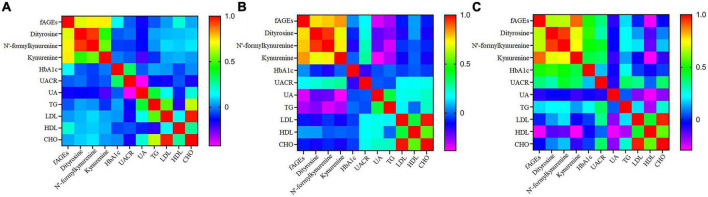
The heatmap of correlation coefficient of fluorescent advanced glycation end products (fAGEs), dityrosine, N’-formylkynurenine and kynurenine, HbA1c, UACR, UA, TG, LDL, HDL, and CHO in patients with diabetes duration ≤5 years **(A)**, 5–10 years **(B)**, and ≥10 years **(C)**.

In order to investigate whether there was a relationship between clinical parameters (HbA1c, UACR, UA, TG, LDL, HDL, and CHO) and serum fAGEs, a correlation analysis was performed in patients with different durations of diabetics. Compared to the patients with diabetes duration <10 years (Figures 3A, B), it was worth noting that a significant correlation was observed between HbA1c and fAGEs or POPs in patients with diabetes duration ≥10 years (Figure 3C). Furthermore, a similar result was also observed between UACR and fAGEs or POPs (Figure 3C). These findings indicated that the increase of fAGEs and protein oxidation products might lead to higher concentrations of HbA1c and UACR in patients with diabetes duration ≥10 years.

### 3.4 Effect of smoking on the formation of fAGEs in patients with T2DM

**FIGURE 4:**
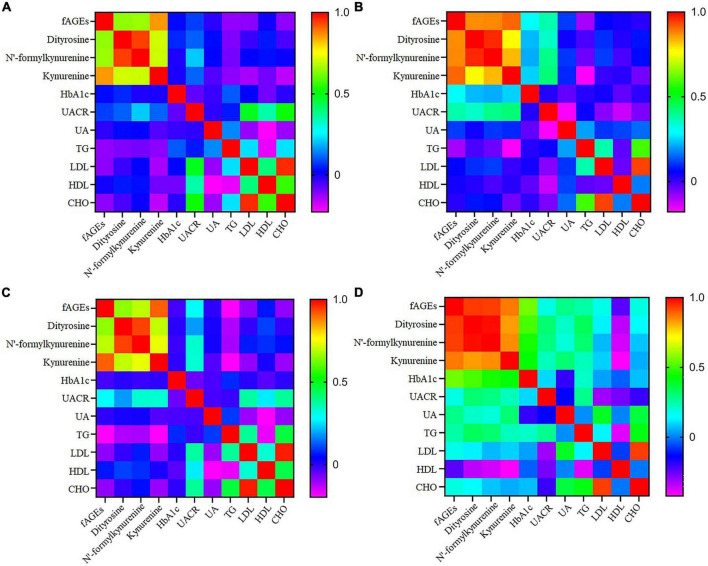
The heatmap of correlation coefficient of fluorescent advanced glycation end products (fAGEs), dityrosine, N’-formylkynurenine and kynurenine, HbA1c, UACR, UA, TG, LDL, HDL, and CHO in T2DM with non-smokers **(A)**, smokers **(B)**, CIMT < 1 **(C)**, and CIMT ≥ 1 **(D)**.

Effect of smoking on the generation of fAGEs was investigated in patients with T2DM. As presented in Table 3, there was no significant difference in the amounts of serum fAGEs and POPs in patients with diabetes duration ≤5 years. In addition, in patients with diabetes duration >5 years, intensities of fAGEs and POPs in smokers with T2DM were significant higher compared to non-smokers with T2DM (Table 3). To further elucidate the reason for increasing fluorescence intensity of AGEs in smokers with T2DM, the relationship between smoking and fAGEs is also evaluated. Compared to the no smoking patients (Figure 4A), a significant correlation was found between fluorescence intensity of AGEs, POPs, and HbA1c or UACR in the smoking patients (Figure 4B). Additionally, the fluorescence intensity of AGEs in smoking patients with diabetes duration ≥10 years was higher compared to the smoking patients with diabetes duration <10 years (Table 3).

**TABLE 3:** Fluorescence intensities of advanced glycation end products (AGEs) and protein oxidation products (POPs) in smokers and non-smokers with type 2 diabetes mellitus (T2DM).

	fAGEs**	Dityrosine**	N’-formylkynurenine**	Kynurenine**
Healthy subjects (*n* = 30)*				
Smokers (*n* = 6)	^BC^21.7 ± 4.4^a^	^B^24.9 ± 5.3^a^	^B^30.1 ± 5.5^a^	^C^24.4 ± 4.8^a^
Non-smokers (*n* = 24)	^C^16.7 ± 1.4^a^	^B^18.4 ± 1.9^a^	^B^23.2 ± 2.1^a^	^C^23.4 ± 1.7^a^
Diabetes duration ≤5 years (*n* = 49)*				
Smokers (*n* = 19)	^C^19.6 ± 2.5^a^	^B^20.7 ± 3.0^a^	^B^22.3 ± 3.1^a^	^C^28.7 ± 2.7^a^
Non-smokers (n = 30)	^BC^19.8 ± 1.2^a^	^B^18.0 ± 1.7^a^	^B^20.0 ± 2.0^a^	^B^29.8 ± 1.5^a^
Diabetes duration 5–10 years (*n* = 33)*				
Smokers (*n* = 10)	^B^29.1 ± 3.4^a^	^B^27.9 ± 4.1^a^	^B^32.8 ± 4.2^a^	^B^42.0 ± 3.7^a^
Non-smokers (*n* = 23)	^B^22.8 ± 1.4^B^	^B^20.3 ± 2.0^B^	^B^23.0 ± 2.2^B^	^B^31.7 ± 1.8^B^
Diabetes duration ≥10 years (*n* = 49)*				
Smokers (*n* = 14)	^a^42.7 ± 2.9^a^	^a^39.5 ± 3.5^a^	^a^44.1 ± 3.6^a^	^a^52.3 ± 3.1^a^
Non-smokers (*n* = 35)	^a^31.6 ± 1.1^B^	^a^30.7 ± 1.6^a^	^a^33.9 ± 1.8^a^	^a^40.2 ± 1.4^B^

*Different letters (a, b) in patients with the same diabetes duration (column) indicate significant differences (*P* < 0.05).

**Different letters (A–C) in smokers or non-smokers with T2DM indicate significant differences (*P* < 0.05).

### 3.5 Correlation between fAGEs and CIMT in patients with T2DM

In order to research the effect of fAGEs on the increment of CIMT, the intensities of fAGEs and protein oxidation products (dityrosine, N’-formylkynurenine, and kynurenine) were investigated in patients with CIMT ≥1 mm and CIMT <1 mm. As presented in Table 4, compared with patients (CIMT < 1 mm), a significant increase of fAGEs was observed in patients (CIMT ≥ 1 mm) with diabetes duration ≥10 years. There was no significant difference in the amounts of fAGEs in patients (diabetes duration <10 years). Besides, in order to investigate whether there was a relationship between serum fAGEs formation and HbA1c, a correlation analysis was performed in patients with T2DM. As shown in Figure 4, compared to the patients with CIMT <1 mm (Figure 4C), a significant correlation was observed between HbA1c and fluorescence intensities of AGEs or POPs in patients with CIMT ≥1 mm (Figure 4D). Moreover, a significant increase of fAGEs was found in patients with CIMT >1 compared to the patients with CIMT <1 (diabetes duration ≥10 years) (Table 4).

**TABLE 4:** Fluorescence intensities of advanced glycation end products (AGEs) and protein oxidation products (POPs) in patients with carotid intima media thickness (CIMT) ≥1 and <1 mm.

	fAGEs**	Dityrosine**	N’-formylkynurenine**	Kynurenine**
Healthy subjects (*n* = 30)*				
CIMT ≥ 1 mm (*n* = 0)	–	–	–	–
CIMT < 1 mm (*n* = 30)	^C^17.7 ± 1.3	^B^19.7 ± 1.8	^B^24.6 ± 2.1	^BC^32.4 ± 2.7
Diabetes duration ≤ 5 years (*n* = 49)*				
CIMT ≥ 1 mm (*n* = 6)	^B^20.2 ± 2.4^a^	^B^18.8 ± 3.2^a^	^B^21.9 ± 3.5^a^	^B^36.6 ± 3.1^a^
CIMT < 1 mm (*n* = 43)	^C^19.6 ± 1.1^a^	^B^19.1 ± 1.5^a^	^B^20.8 ± 1.7^a^	^C^28.3 ± 2.3^B^
Diabetes duration 5-10 years (n == 33)*				
CIMT ≥ 1 mm (*n* = 6)	^B^26.4 ± 2.5^a^	^B^23.1 ± 3.3^a^	^B^26.8 ± 4.1^a^	^AB^38.2 ± 3.9^a^
CIMT < 1 mm (*n* = 27)	^B^24.4 ± 1.4^a^	^B^22.5 ± 1.9^a^	^B^25.8 ± 2.2^a^	^B^36.0 ± 2.9^a^
Diabetes duration ≥ 10 years (n = 49)*				
CIMT ≥ 1 mm (*n* = 11)	^a^46.4 ± 3.6^a^	^a^41.8 ± 4.6^a^	^a^45.1 ± 5.0^a^	^a^51.1 ± 4.0^a^
CIMT < 1 mm (*n* = 38)	^a^31.4 ± 1.2^B^	^a^30.8 ± 1.6^B^	^a^34.4 ± 1.8^B^	^a^48.3 ± 2.4^a^

*Different letters (a, b) in patients with the same diabetes duration (column) indicate significant differences (*P* < 0.05).

**Different letters (A–C) in patients with CIMT ≥ 1 mm or CIMT < 1 mm indicate significant differences (*P* < 0.05).

## 4 Discussion

As indicated above, the present study suggested that duration of diabetics could significantly affect protein oxidation. Generally, carbonylation was recognized as one of the most important oxidative modifications of protein (32), and the oxidative degree of lipid was evaluated by levels of malondialdehyde (MDA) (33). Pan et al. (34) investigated the relationship between the oxidative stress status and diabetes complications in patients with T2DM, which found that diabetes duration significant positively correlated with MDA, advanced oxidation protein products and protein carbonyl (*P* < 0.05). These findings discussed here indicated oxidative stress was correlated to diabetes duration. Increased oxidative stress in patients with T2DM could induce the oxidation of protein (35, 36), resulting in higher levels of protein oxidation products in patients with long-duration diabetics. Furthermore, duration of diabetics could significantly promote the formation of fAGEs. This result seems to be reasonable because protein oxidation products was immediate precursor of fAGEs, higher levels of protein oxidation products would promote the formation of fAGEs (37). Usually, fAGEs can be formed via the Maillard reaction or lipid oxidation pathway (38). In this study, increased oxidative stress in patients with T2DM could induce the formation of free radicals (such as hydroxyl radical, superoxide radical and cross-linked radical cation) and lipid oxidation, which promoted the generation of fAGEs (39). The fluorescence intensities of AGEs and protein oxidation products increased with the increasing duration of diabetics (*P* < 0.05), probably due to the increased oxidative stress and lipid oxidation. Therefore, it was reasonable that fAGEs in patients with diabetes duration ≥10 years was higher compared to the patients with diabetes duration <10 years. Diabetes duration could significantly promote the formation of serum fAGEs and protein oxidation products.

Changes in the physiology or pathology of patients could be reflected in clinical parameters. For example, HbA1c is an important indicator of long-term glycemic control, which could reflect the cumulative glycemic history of the preceding 2–3 months (40). Glycation is also a major cause of spontaneous damage to extracellular and cellular proteins of living organisms (41). The present study indicated that higher levels of fAGEs and protein oxidation products might promote the increase of HbA1c level. The result was consistent with previous findings that correlation coefficient between AGEs and HbA1c in patients with T2DM was 0.661 (42). In general, the formation of AGEs contain three main steps: (1) carbonyl group of lipid oxidation products or reducing sugar react with protein to form Schiff’s base, which becomes to Amadori products after rearrangement; (2) Amadori products dehydration and rearrangement generates highly reactive dicarbonyl compounds, such as 3-deoxyglucosone (3-DG), glyoxal (GO), and methylglyoxal (MGO); and (3) these carbonyl compounds react with arginine and lysine residues of proteins to form a stable AGEs (8, 43, 44). Similarly, HbA1c is also the product of Amadori rearrangement that formed during the process of glycation (45). Therefore, higher levels of HbA1c probably due to increased overall protein glycation reactions (46). Besides, Chao et al. (42) also reported that AGEs might enhance glycation reactions of hemoglobin, which subsequently enhance the formation of HbA1c in patients with T2DM. The fluorescence intensity of AGEs could be considered as a marker of long-term glycemic control in patients with T2DM.

Not only is smoking a risk factor for developing diabetes (47), smoking also affects the formation of AGEs (48). Therefore, the impact of diabetes and some general habits, such as smoking, on the formation of fAGEs also needs to be further investigated. The present study indicated fAGE levels in smokers were significantly higher than those in non-smokers (diabetes duration ≥10 years). This result meant that smoking might contribute to levels of fAGEs in patients with T2DM. Cerami et al. (48) reported that reactive glycation products were present in aqueous extracts of tobacco and in tobacco smoke in a form that could rapidly react with proteins to form AGEs, resulting in a significant increase in serum AGEs contents in smokers. Besides, AGEs in tobacco and tobacco smoke were diet-derived AGEs, and dietary AGEs might be released into the blood stream or directly gain entry into the systemic circulation (5). Therefore, consumption of tobacco diet resulted in increase of plasma levels of AGEs. Furthermore, smoking and diabetes duration might have a synergistic effect on the formation of fAGEs in patients with T2DM, as evidence by the results of correlation analysis in patients with long-duration diabetics (≥10 years). Facchini et al. (49) investigated that relationship between insulin resistance and cigarette smoking, which suggested that chronic cigarette smokers were insulin resistant and hyperinsulinaemic compared with a matched group of non-smokers. This result might help to explain why smoking could increase levels of HbA1c and fAGEs in patients with T2DM. The results are consistent with a previous report which demonstrated that smoking rates had a good correlation with HbA1c levels in patients with T2DM (50).

Diabetes complications have been paid attention because of harmful effects on human health (51). Carotid atherosclerosis, as one of the complications of diabetes, has been widely studied (26). CIMT is the most often used for carotid atherosclerosis evaluation in clinical trials (52). The present results demonstrated that fAGEs could significantly affect the increase of CIMT in patients with diabetes duration ≥10 years. It should be noted that HbA1c in patients with diabetes duration ≥10 years was higher than those in patients with diabetes duration <10 years (Table 1) (*P* < 0.05). Indyk et al. (45) reported that fAGEs was generated through various chemical pathways, such as Schiff’s base and Amadori rearrangement, which might promote the increment of HbA1c level (53). Chao et al. (42) has been documented that fAGEs could enhance glycation reactions of hemoglobin, which promote the formation of HbA1c in patients with T2DM. James et al. (54) also reported that glycosylation of hemoglobin could alter nitric oxide binding to hemoglobin thiols and impair vasodilatation, which lead to an increase in CIMT. Higher levels of fAGEs might cause the increase of HbA1c, which further lead to an increase in CIMT (55). Therefore, the fluorescence intensities of AGEs could be considered as a marker for carotid atherosclerosis.

## 5 Conclusion

In summary, the results of this study demonstrate the relationships between serum fAGEs levels and CIMT or clinical parameters in patients with T2DM. The fluorescence intensities of AGEs and POPs increased with the increasing duration of diabetics. Diabetes duration and smoking markedly promoted the accumulation of fAGEs and POPs. Higher concentrations of fAGEs might cause the increase of HbA1c and UACR levels. A continued increase in fluorescence intensity of AGEs might cause the increase of CIMT in patients with T2DM. These findings reflected that increasing fAGEs might enrich circulating AGEs levels and contribute to impair vasodilatation progression in patients with T2DM, which subsequently lead to a significant increment in CIMT. Therefore, the fluorescence intensity of AGEs could be considered as a marker for the duration of diabetics and carotid atherosclerosis. This work might be helpful to advance our knowledge on the overall risk of complications in patients with T2DM.

## Data availability statement

The raw data supporting the conclusions of this article will be made available by the authors, without undue reservation.

## Ethics statement

The studies involving human participants were reviewed and approved by Ethics Committee of Shanxi Provincial People’s Hospital (approval no. 2022023). The patients/participants provided their written informed consent to participate in this study.

## Author contributions

RL: conceptualization, funding acquisition, and writing—original draft. MZ and LX: investigation. JL and PY: data curation. ML: project administration. JQ: writing—review and editing. All authors contributed to the article and approved the submitted version.

## References

[1] Zhu Z Fang R Huang M Wei Y Zhou G. Oxidation combined with Maillard reaction induced free and protein-bound N^ε^ -carboxymethyllysine and N^ε^ -carboxyethyllysine formation during braised chicken processing. *Food Sci Hum Wellness.* (2020) 9:383–93. 10.1016/j.fshw.2020.05.013

[2] Sergi D Boulestin H Campbell F Williams L. The role of dietary advanced glycation end products in metabolic dysfunction. *Mol Nutr Food Res.* (2021) 65:e1900934. 10.1002/mnfr.201900934 32246887

[3] Yu L Li Y Gao C Yang Y Zeng M Chen J. Nε-carboxymethyl-lysine and Nε-carboxyethyl-lysine contents in commercial meat products. *Food Res Int.* (2022) 155:111048. 10.1016/j.foodres.2022.111048 35400433

[4] Luevano-Contreras C Chapman-Novakofski K. Dietary advanced glycation end products and aging. *Nutrients.* (2010) 2:1247–65. 10.3390/nu2121247 22254007 PMC3257625

[5] Uribarri J Cai W Sandu O Peppa M Goldberg T Vlassara H. Diet-derived advanced glycation end products are major contributors to the body’s AGE pool and induce inflammation in healthy subjects. *Ann N Y Acad Sci.* (2005) 1043:461–6. 10.1196/annals.1333.052 16037267

[6] Wu X Zhang D Wang Y Tan Y Yu X Zhao YY. AGE/RAGE in diabetic kidney disease and ageing kidney. *Free Radic Biol Med.* (2021) 171:260–71. 10.1016/j.freeradbiomed.2021.05.025 34019934

[7] Parveen A Sultana R Lee S Kim T Kim S. Phytochemicals against anti-diabetic complications: targeting the advanced glycation end product signaling pathway. *Arch Pharm Res.* (2021) 44:378–401. 10.1007/s12272-021-01323-9 33837513

[8] Baynes J Thorpe S. Glycoxidation and lipoxidation in atherogenesis. *Free Radic Biol Med.* (2000) 28:1708–16. 10.1016/s0891-5849(00)00228-8 10946212

[9] Sharma A Weber D Raupbach J Dakal T Fließbach K Ramirez A Advanced glycation end products and protein carbonyl levels in plasma reveal sex-specific differences in Parkinson’s and Alzheimer’s disease. *Redox Biol.* (2020) 34:101546. 10.1016/j.redox.2020.101546 32460130 PMC7251371

[10] Menini S Iacobini C de Latouliere L Manni I Ionta V Blasetti Fantauzzi C The advanced glycation end-product N^ε^ -carboxymethyllysine promotes progression of pancreatic cancer: implications for diabetes-associated risk and its prevention. *J Pathol.* (2018) 245:197–208. 10.1002/path.5072 29533466

[11] Garay-Sevilla M Rojas A Portero-Otin M Uribarri J. Dietary AGEs as exogenous boosters of inflammation. *Nutrients.* (2021) 13:2802. 10.3390/nu13082802 34444961 PMC8401706

[12] Nie C Li Y Qian H Ying H Wang L. Advanced glycation end products in food and their effects on intestinal tract. *Crit Rev Food Sci Nutr.* (2022) 62:3103–15. 10.1080/10408398.2020.1863904 33356474

[13] Davis K Prasad C Vijayagopal P Juma S Imrhan V. Advanced glycation end products, inflammation, and chronic metabolic diseases: links in a chain? *Crit Rev Food Sci Nutr.* (2016) 56:989–98. 10.1080/10408398.2012.744738 25259686

[14] van Dongen K Kappetein L Miro Estruch I Belzer C Beekmann K Rietjens I. Differences in kinetics and dynamics of endogenous versus exogenous advanced glycation end products (AGEs) and their precursors. *Food Chem Toxicol.* (2022) 164:112987. 10.1016/j.fct.2022.112987 35398182

[15] Koska J Saremi A Howell S Bahn G De Courten B Ginsberg H Advanced glycation end products, oxidation products, and incident cardiovascular events in patients with type 2 diabetes. *Diabetes Care.* (2018) 41:570–6. 10.2337/dc17-1740 29208654 PMC5829965

[16] Akram Z Alqahtani F Alqahtani M Al-Kheraif A Javed F. Levels of advanced glycation end products in gingival crevicular fluid of chronic periodontitis patients with and without type-2 diabetes mellitus. *J Periodontol.* (2020) 91:396–402. 10.1002/JPER.19-0209 31389020

[17] Cai W Ramdas M Zhu L Chen X Striker G Vlassara H. Oral advanced glycation endproducts (AGEs) promote insulin resistance and diabetes by depleting the antioxidant defenses AGE receptor-1 and sirtuin 1. *Proc Natl Acad Sci U.S.A.* (2012) 109:15888–93. 10.1073/pnas.1205847109 22908267 PMC3465382

[18] Tan K Shiu S Wong Y Tam X. Serum advanced glycation end products (AGEs) are associated with insulin resistance. *Diabetes Metab Res Rev.* (2011) 27:488–92. 10.1002/dmrr.1188 21337488

[19] Uribarri J Cai W Ramdas M Goodman S Pyzik R Chen X Restriction of advanced glycation end products improves insulin resistance in human type 2 diabetes: potential role of AGER1 and SIRT1. *Diabetes Care.* (2011) 34:1610–6. 10.2337/dc11-0091 21709297 PMC3120204

[20] Hellwig M Henle T. Baking, ageing, diabetes: a short history of the Maillard reaction. *Angew Chem Int Ed Engl.* (2014) 53:10316–29. 10.1002/anie.201308808 25044982

[21] Katz J Yoon T Mao S Lamont R Caudle R. Expression of the receptor of advanced glycation end products in the gingival tissue of smokers with generalized periodontal disease and after nornicotine induction in primary gingival epithelial cells. *J Periodontol.* (2007) 78:736–41. 10.1902/jop.2007.060381 17397323

[22] Rungratanawanich W Qu Y Wang X Essa M Song B. Advanced glycation end products (AGEs) and other adducts in aging-related diseases and alcohol-mediated tissue injury. *Exp Mol Med.* (2021) 53:168–88. 10.1038/s12276-021-00561-7 33568752 PMC8080618

[23] Yu L Li Y Yang Y Guo C Li M. Inhibitory effects of curcumin and piperine on fluorescent advanced glycation end products formation in a bovine serum albumin–fructose model. *Int J Food Sci Tech.* (2022) 57:4646–55. 10.1111/ijfs.15804

[24] Perrone A Giovino A Benny J Martinelli F. Advanced glycation end products (AGEs): biochemistry, signaling, analytical methods, and epigenetic effects. *Oxid Med Cell Longev.* (2020) 2020:3818196. 10.1155/2020/3818196 32256950 PMC7104326

[25] Ou J Huang J Wang M Ou S. Effect of rosmarinic acid and carnosic acid on AGEs formation in vitro. *Food Chem.* (2017) 221:1057–61. 10.1016/j.foodchem.2016.11.056 27979058

[26] Jud P Sourij H. Therapeutic options to reduce advanced glycation end products in patients with diabetes mellitus: a review. *Diabetes Res Clin Pract.* (2019) 148:54–63. 10.1016/j.diabres.2018.11.016 30500546

[27] Thevarajah M Nadzimah M Chew Y. Interference of hemoglobinA1c (HbA1c) detection using ion-exchange high performance liquid chromatography (HPLC) method by clinically silent hemoglobin variant in University Malaya Medical Centre (UMMC)–a case report. *Clin Biochem.* (2009) 42:430–4. 10.1016/j.clinbiochem.2008.10.015 19026622

[28] Jun J Kang H Hwang Y Ahn K Chung H Jeong I. The association between lipoprotein (a) and carotid atherosclerosis in patients with type 2 diabetes without pre-existing cardiovascular disease: a cross-sectional study. *Diabetes Res Clin Pract.* (2021) 171:108622. 10.1016/j.diabres.2020.108622 33316308

[29] Lee H Cho Y Choi Y Huh B Lee B Kang E Non-alcoholic steatohepatitis and progression of carotid atherosclerosis in patients with type 2 diabetes: a Korean cohort study. *Cardiovasc Diabetol.* (2020) 19:81. 10.1186/s12933-020-01064-x 32534588 PMC7293796

[30] Cambray S Ibarz M Bermudez-Lopez M Marti-Antonio M Bozic M Fernandez E Magnesium levels modify the effect of lipid parameters on carotid intima media thickness. *Nutrients.* (2020) 12:2631. 10.3390/nu12092631 32872319 PMC7551902

[31] Ferrer E Alegría A Farré R Clemente G Calvo C. Fluorescence, browning index, and color in infant formulas during storage. *J Agric Food Chem.* (2005) 53:4911–7. 10.1021/jf0403585 15941335

[32] Villaverde A Estévez M. Carbonylation of myofibrillar proteins through the maillard pathway: effect of reducing sugars and reaction temperature. *J Agric Food Chem.* (2013) 61:3140–7. 10.1021/jf305451p 23438261

[33] Adnan M Amin M Uddin M Hussain M Sarwar M Hossain M Increased concentration of serum MDA, decreased antioxidants and altered trace elements and macro-minerals are linked to obesity among Bangladeshi population. *Diabetes Metab Syndr.* (2019) 13:933–8. 10.1016/j.dsx.2018.12.022 31336547

[34] Pan H Zhang L Guo M Sui H Li H Wu W The oxidative stress status in diabetes mellitus and diabetic nephropathy. *Acta Diabetol.* (2010) 47:71–6. 10.1007/s00592-009-0128-1 19475334

[35] Bhatia S Shukla R Venkata Madhu S Kaur Gambhir J Madhava Prabhu K. Antioxidant status, lipid peroxidation and nitric oxide end products in patients of type 2 diabetes mellitus with nephropathy. *Clin Biochem.* (2003) 36:557–62. 10.1016/s0009-9120(03)00094-8 14563450

[36] Dursun E Timur M Dursun B Süleymanlar G Ozben T. Protein oxidation in type 2 diabetic patients on hemodialysis. *J Diabetes Complications.* (2005) 19:142–6. 10.1016/j.jdiacomp.2004.11.001 15866059

[37] Yu L He Z Zeng M Yang Y Chen J. Effect of oxidation and hydrolysis of porcine myofibrillar protein on N^ε^ -carboxymethyl-lysine formation in model systems. *Int J Food Sci Tech.* (2021) 56:3076–84. 10.1111/ijfs.14951

[38] Poulsen M Hedegaard R Andersen J de Courten B Bügel S Nielsen J Advanced glycation endproducts in food and their effects on health. *Food Chem Toxicol.* (2013) 60:10–37. 10.1016/j.fct.2013.06.052 23867544

[39] Peng X Ma J Chen F Wang M. Naturally occurring inhibitors against the formation of advanced glycation end-products. *Food Funct.* (2011) 2:289–301. 10.1039/c1fo10034c 21779567

[40] Wei Q Chen Y Cao B Ou R Zhang L Hou Y Blood hemoglobin A1c levels and amyotrophic lateral sclerosis survival. *Mol Neurodegener.* (2017) 12:69. 10.1186/s13024-017-0211-y 28934974 PMC5609007

[41] Deluyker D Evens L Bito V. Advanced glycation end products (AGEs) and cardiovascular dysfunction: focus on high molecular weight AGEs. *Amino Acids.* (2017) 49:1535–41. 10.1007/s00726-017-2464-8 28710551

[42] Chao P Huang C Hsu C Yin M Guo Y. Association of dietary AGEs with circulating AGEs, glycated LDL, IL-1α and MCP-1 levels in type 2 diabetic patients. *Eur J Nutr.* (2010) 49:429–34. 10.1007/s00394-010-0101-3 20229096

[43] Ames J. Determination of N epsilon-(carboxymethyl)lysine in foods and related systems. *Ann N Y Acad Sci.* (2008) 1126:20–4. 10.1196/annals.1433.030 18448791

[44] Erbersdobler H Somoza V. Forty years of furosine - forty years of using Maillard reaction products as indicators of the nutritional quality of foods. *Mol Nutr Food Res.* (2007) 51:423–30. 10.1002/mnfr.200600154 17390403

[45] Indyk D Bronowicka-Szydełko A Gamian A Kuzan A. Advanced glycation end products and their receptors in serum of patients with type 2 diabetes. *Sci Rep.* (2021) 11:13264. 10.1038/s41598-021-92630-0 34168187 PMC8225908

[46] Sherwani S Khan H Ekhzaimy A Masood A Sakharkar M. Significance of HbA1c test in diagnosis and prognosis of diabetic patients. *Biomark Insights.* (2016) 11:95–104. 10.4137/BMI.S38440 27398023 PMC4933534

[47] Śliwińska-Mossoń M Milnerowicz H. The impact of smoking on the development of diabetes and its complications. *Diab Vasc Dis Res.* (2017) 14:265–76. 10.1177/1479164117701876 28393534

[48] Cerami C Founds H Nicholl I Mitsuhashi T Giordano D Vanpatten S Tobacco smoke is a source of toxic reactive glycation products. *Proc Natl Acad Sci U.S.A.* (1997) 94:13915–20. 10.1073/pnas.94.25.13915 9391127 PMC28407

[49] Facchini F Hollenbeck C Jeppesen J Chen Y Reaven G. Insulin resistance and cigarette smoking. *Lancet.* (1992) 339:1128–30. 10.1016/0140-6736(92)90730-q 1349365

[50] Katon W von Korff M Ciechanowski P Russo J Lin E Simon G Behavioral and clinical factors associated with depression among individuals with diabetes. *Diabetes Care.* (2004) 27:914–20. 10.2337/diacare.27.4.914 15047648

[51] Karami H Shirvani Shiri M Rezapour A Sarvari Mehrabadi R Afshari S. The association between diabetic complications and health-related quality of life in patients with type 2 diabetes: a cross-sectional study from Iran. *Qual Life Res.* (2021) 30:1963–74. 10.1007/s11136-021-02792-7 33900519

[52] Touboul P Grobbee D den Ruijter H. Assessment of subclinical atherosclerosis by carotid intima media thickness: technical issues. *Eur J Prev Cardiol.* (2012) 19:18–24. 10.1177/2047487312448990 22801066

[53] Meerwaldt R Links T Zeebregts C Tio R Hillebrands J Smit A. The clinical relevance of assessing advanced glycation endproducts accumulation in diabetes. *Cardiovasc Diabetol.* (2008) 7:29. 10.1186/1475-2840-7-29 18840258 PMC2569910

[54] James P Lang D Tufnell-Barret T Milsom A Frenneaux M. Vasorelaxation by red blood cells and impairment in diabetes: reduced nitric oxide and oxygen delivery by glycated hemoglobin. *Circ Res.* (2004) 94:976–83. 10.1161/01.RES.0000122044.21787.01 14963010

[55] Saba L Ikeda N Deidda M Araki T Molinari F Meiburger K Association of automated carotid IMT measurement and HbA1c in Japanese patients with coronary artery disease. *Diabetes Res Clin Pract.* (2013) 100:348–53. 10.1016/j.diabres.2013.03.032 23611290

