# Supplementary material for: Fluorescent advanced glycation end products in type 2 diabetes and its association with diabetes duration, hemoglobin A1c, and diabetic complications
Source: Front Nutr. 2022 Dec 15;9:1083872. doi: 10.3389/fnut.2022.1083872 (PMC9797537; doi:10.3389/fnut.2022.1083872)
Supplement: Supplementary file 1 [file Table_1.DOC]

**Supplemental table 1** Pearson's correlation coefficient (r) of fAGEs, protein oxidation products (OPs1, OPs2, OPs3), HbA1c, UACR, UA, TG, LDL, HDL and CHO in patients with diabetes duration ≤ 5 years.^*^

|  | fAGEs | OPs1 | OPs2 | OPs3 | HBA1c | UACR | UA | TG | LDL | HDL | CHO |
| --- | --- | --- | --- | --- | --- | --- | --- | --- | --- | --- | --- |
| fAGEs | 1.000 | 0.668 | 0.717 | 0.728 | 0.120 | 0.035 | -0.159 | -0.018 | -0.036 | 0.149 | 0.003 |
| OPs1 | 0.668 | 1.000 | 0.930 | 0.475 | -0.048 | 0.019 | -0.145 | 0.020 | 0.038 | -0.002 | 0.057 |
| OPs2 | 0.717 | 0.930 | 1.000 | 0.533 | -0.116 | 0.042 | -0.132 | -0.005 | 0.064 | -0.016 | 0.075 |
| OPs3 | 0.728 | 0.475 | 0.533 | 1.000 | 0.033 | 0.062 | -0.221 | -0.105 | -0.026 | -0.025 | -0.056 |
| HBA1c | 0.120 | -0.048 | -0.116 | 0.033 | 1.000 | 0.195 | -0.040 | -0.001 | 0.079 | -0.175 | -0.002 |
| UACR | 0.035 | 0.019 | 0.042 | 0.062 | 0.195 | 1.000 | -0.425 | -0.150 | -0.211 | -0.115 | -0.255 |
| UA | -0.159 | -0.145 | -0.132 | -0.221 | -0.040 | -0.425 | 1.000 | 0.378 | 0.171 | -0.106 | 0.198 |
| TG | -0.019 | 0.020 | -0.005 | -0.105 | -0.001 | -0.150 | 0.378 | 1.000 | 0.426 | -0.032 | 0.607 |
| LDL | -0.036 | 0.038 | 0.064 | -0.026 | 0.079 | -0.211 | 0.171 | 0.426 | 1.000 | -0.046 | 0.917 |
| HDL | 0.149 | -0.002 | -0.016 | -0.025 | -0.175 | -0.115 | -0.106 | -0.032 | -0.046 | 1.000 | 0.194 |
| CHO | 0.003 | 0.057 | 0.075 | -0.056 | -0.002 | -0.255 | 0.198 | 0.607 | 0.917 | 0.194 | 1.000 |

^*^ fAGEs, Fluorescent advanced glycation end products; OPs1, dityrosine; OPs2, N'-formylkynurenine; OPs3, kynurenine; HbA1c, hemoglobin A1C; UACR, urinary albumin/creatinine ratio; UA, serum uric acid; TG, triglyceride; LDL, low-density lipoprotein; HDL, high-density lipoprotein, CHO, cholesterol.

**Supplemental table 2 Pearson's correlation coefficient (r) of fAGEs, protein oxidation products (OPs1, OPs2, OPs3), HbA1c, UACR, UA, TG, LDL, HDL and CHO in patients with diabetes duration 5-10 years.^*^**

|  | fAGEs | OPs1 | OPs2 | OPs3 | HBA1c | UACR | UA | TG | LDL | HDL | CHO |
| --- | --- | --- | --- | --- | --- | --- | --- | --- | --- | --- | --- |
| fAGEs | 1.000 | 0.778 | 0.801 | 0.840 | 0.093 | 0.142 | -0.253 | -0.300 | -0.150 | 0.070 | -0.149 |
| OPs1 | 0.778 | 1.000 | 0.943 | 0.688 | -0.047 | 0.200 | -0.140 | -0.251 | -0.176 | 0.062 | -0.168 |
| OPs2 | 0.801 | 0.943 | 1.000 | 0.735 | -0.107 | 0.227 | -0.115 | -0.330 | -0.108 | -0.003 | -0.126 |
| OPs3 | 0.840 | 0.688 | 0.735 | 1.000 | 0.089 | 0.252 | -0.326 | -0.327 | -0.216 | 0.013 | -0.219 |
| HBA1c | 0.093 | -0.048 | -0.107 | 0.089 | 1.000 | -0.173 | -0.029 | -0.031 | -0.119 | 0.003 | -0.118 |
| UACR | 0.142 | 0.200 | 0.227 | 0.252 | -0.173 | 1.000 | -0.018 | -0.027 | 0.164 | 0.182 | 0.200 |
| UA | -0.253 | -0.140 | -0.115 | -0.326 | -0.029 | -0.017 | 1.000 | 0.370 | 0.167 | 0.201 | 0.201 |
| TG | -0.300 | -0.251 | -0.330 | -0.327 | -0.031 | -0.027 | 0.370 | 1.000 | 0.195 | -0.008 | 0.269 |
| LDL | -0.150 | -0.176 | -0.108 | -0.216 | -0.119 | 0.164 | 0.167 | 0.195 | 1.000 | 0.358 | 0.978 |
| HDL | 0.070 | 0.062 | -0.003 | 0.013 | 0.003 | 0.182 | 0.201 | -0.008 | 0.358 | 1.000 | 0.503 |
| CHO | -0.149 | -0.168 | -0.126 | -0.219 | -0.118 | 0.200 | 0.201 | 0.269 | 0.978 | 0.503 | 1.000 |

^*^ fAGEs, Fluorescent advanced glycation end products; OPs1, dityrosine; OPs2, N'-formylkynurenine; OPs3, kynurenine; HbA1c, hemoglobin A1C; UACR, urinary albumin/creatinine ratio; UA, serum uric acid; TG, triglyceride; LDL, low-density lipoprotein; HDL, high-density lipoprotein, CHO, cholesterol.

**Supplemental table 3 Pearson's correlation coefficient (r) of fAGEs, protein oxidation products (OPs1, OPs2, OPs3), HbA1c, UACR, UA, TG, LDL, HDL and CHO in patients with diabetes duration ≥ 10 years.^*^**

|  | fAGEs | OPs1 | OPs2 | OPs3 | HBA1c | UACR | UA | TG | LDL | HDL | CHO |
| --- | --- | --- | --- | --- | --- | --- | --- | --- | --- | --- | --- |
| fAGEs | 1.000 | 0.659 | 0.656 | 0.894 | 0.505 | 0.370 | -0.078 | 0.175 | 0.049 | -0.197 | 0.008 |
| OPs1 | 0.659 | 1.000 | 0.939 | 0.715 | 0.526 | 0.315 | -0.031 | 0.228 | 0.137 | -0.069 | 0.105 |
| OPs2 | 0.656 | 0.939 | 1.000 | 0.732 | 0.489 | 0.443 | -0.027 | 0.252 | 0.190 | -0.098 | 0.149 |
| OPs3 | 0.894 | 0.715 | 0.732 | 1.000 | 0.422 | 0.390 | -0.090 | 0.126 | -0.016 | -0.235 | -0.064 |
| HBA1c | 0.505 | 0.526 | 0.489 | 0.422 | 1.000 | 0.158 | -0.048 | 0.252 | 0.043 | -0.013 | 0.037 |
| UACR | 0.370 | 0.315 | 0.443 | 0.390 | 0.158 | 1.000 | -0.070 | 0.386 | 0.344 | 0.069 | 0.386 |
| UA | -0.078 | -0.031 | -0.027 | -0.090 | -0.048 | -0.070 | 1.000 | 0.062 | -0.171 | -0.270 | -0.173 |
| TG | 0.175 | 0.228 | 0.252 | 0.126 | 0.252 | 0.386 | 0.062 | 1.000 | 0.155 | -0.222 | 0.245 |
| LDL | 0.049 | 0.137 | 0.190 | -0.016 | 0.043 | 0.344 | -0.171 | 0.155 | 1.000 | 0.474 | 0.968 |
| HDL | -0.197 | -0.069 | -0.098 | -0.235 | -0.013 | 0.069 | -0.270 | -0.222 | 0.474 | 1.000 | 0.600 |
| CHO | 0.008 | 0.105 | 0.149 | -0.064 | 0.037 | 0.386 | -0.173 | 0.245 | 0.968 | 0.600 | 1.000 |

^*^ fAGEs, Fluorescent advanced glycation end products; OPs1, dityrosine; OPs2, N'-formylkynurenine; OPs3, kynurenine; HbA1c, hemoglobin A1C; UACR, urinary albumin/creatinine ratio; UA, serum uric acid; TG, triglyceride; LDL, low-density lipoprotein; HDL, high-density lipoprotein, CHO, cholesterol.

**Supplemental table 4 Pearson's correlation coefficient (r) of fAGEs, protein oxidation products (OPs1, OPs2, OPs3), HbA1c, UACR, UA, TG, LDL, HDL and CHO in T2DM with CIMT < 1.^*^**

|  | fAGEs | OPs1 | OPs2 | OPs3 | HBA1c | UACR | UA | TG | LDL | HDL | CHO |
| --- | --- | --- | --- | --- | --- | --- | --- | --- | --- | --- | --- |
| fAGEs | 1.000 | 0.653 | 0.688 | 0.905 | -0.036 | 0.289 | -0.001 | -0.188 | -0.061 | 0.045 | -0.076 |
| OPs1 | 0.653 | 1.000 | 0.938 | 0.698 | -0.013 | 0.188 | 0.024 | -0.124 | 0.005 | 0.061 | -0.008 |
| OPs2 | 0.688 | 0.938 | 1.000 | 0.730 | -0.043 | 0.332 | 0.030 | -0.117 | 0.061 | 0.033 | 0.038 |
| OPs3 | 0.905 | 0.698 | 0.730 | 1.000 | 0.003 | 0.330 | -0.032 | -0.200 | -0.085 | -0.020 | -0.113 |
| HBA1c | -0.036 | -0.013 | -0.043 | 0.003 | 1.000 | -0.070 | -0.024 | 0.054 | 0.030 | -0.102 | -0.010 |
| UACR | 0.289 | 0.188 | 0.332 | 0.330 | -0.070 | 1.000 | -0.024 | -0.022 | 0.348 | 0.220 | 0.355 |
| UA | -0.001 | 0.024 | 0.030 | -0.032 | -0.024 | -0.024 | 1.000 | 0.096 | -0.105 | -0.199 | -0.100 |
| TG | -0.188 | -0.124 | -0.117 | -0.200 | 0.054 | -0.022 | 0.096 | 1.000 | 0.322 | -0.086 | 0.433 |
| LDL | -0.061 | 0.005 | 0.061 | -0.085 | 0.030 | 0.348 | -0.105 | 0.322 | 1.000 | 0.238 | 0.952 |
| HDL | 0.045 | 0.061 | 0.033 | -0.020 | -0.102 | 0.220 | -0.199 | -0.086 | 0.238 | 1.000 | 0.421 |
| CHO | -0.076 | -0.008 | 0.038 | -0.113 | -0.010 | 0.355 | -0.100 | 0.433 | 0.952 | 0.421 | 1.000 |

^*^ fAGEs, Fluorescent advanced glycation end products; OPs1, dityrosine; OPs2, N'-formylkynurenine; OPs3, kynurenine; HbA1c, hemoglobin A1C; UACR, urinary albumin/creatinine ratio; UA, serum uric acid; TG, triglyceride; LDL, low-density lipoprotein; HDL, high-density lipoprotein, CHO, cholesterol; T2DM, type 2 diabetes mellitus; CIMT, carotid intima media thickness.

**Supplemental table 5 Pearson's correlation coefficient (r) of fAGEs, protein oxidation products (OPs1, OPs2, OPs3), HbA1c, UACR, UA, TG, LDL, HDL and CHO in T2DM with CIMT > 1.^*^**

|  | fAGEs | OPs1 | OPs2 | OPs3 | HBA1c | UACR | UA | TG | LDL | HDL | CHO |
| --- | --- | --- | --- | --- | --- | --- | --- | --- | --- | --- | --- |
| fAGEs | 1.000 | 0.937 | 0.932 | 0.867 | 0.551 | 0.184 | 0.288 | 0.254 | 0.225 | -0.064 | 0.214 |
| OPs1 | 0.937 | 1.000 | 0.984 | 0.828 | 0.509 | 0.308 | 0.221 | 0.337 | 0.179 | -0.147 | 0.174 |
| OPs2 | 0.932 | 0.984 | 1.000 | 0.845 | 0.455 | 0.290 | 0.206 | 0.311 | 0.122 | -0.159 | 0.111 |
| OPs3 | 0.867 | 0.828 | 0.845 | 1.000 | 0.434 | 0.230 | 0.313 | 0.234 | 0.151 | -0.202 | 0.087 |
| HBA1c | 0.551 | 0.509 | 0.455 | 0.434 | 1.000 | 0.097 | -0.189 | 0.217 | 0.067 | 0.061 | 0.086 |
| UACR | 0.184 | 0.308 | 0.290 | 0.230 | 0.097 | 1.000 | -0.137 | 0.277 | -0.311 | -0.285 | -0.204 |
| UA | 0.288 | 0.221 | 0.206 | 0.313 | -0.189 | -0.137 | 1.000 | 0.018 | 0.406 | 0.070 | 0.374 |
| TG | 0.254 | 0.337 | 0.311 | 0.234 | 0.217 | 0.276 | 0.018 | 1.000 | 0.122 | -0.388 | 0.402 |
| LDL | 0.225 | 0.179 | 0.122 | 0.152 | 0.067 | -0.311 | 0.406 | 0.122 | 1.000 | 0.019 | 0.930 |
| HDL | -0.064 | -0.147 | -0.159 | -0.202 | 0.061 | -0.285 | 0.069 | -0.388 | 0.0191 | 1.000 | 0.045 |
| CHO | 0.214 | 0.174 | 0.111 | 0.087 | 0.086 | -0.204 | 0.374 | 0.402 | 0.930 | 0.045 | 1.000 |

^*^ fAGEs, Fluorescent advanced glycation end products; OPs1, dityrosine; OPs2, N'-formylkynurenine; OPs3, kynurenine; HbA1c, hemoglobin A1C; UACR, urinary albumin/creatinine ratio; UA, serum uric acid; TG, triglyceride; LDL, low-density lipoprotein; HDL, high-density lipoprotein, CHO, cholesterol; T2DM, type 2 diabetes mellitus; CIMT, carotid intima media thickness.

**Supplemental table 6 Pearson's correlation coefficient (r) of fAGEs, protein oxidation products (OPs1, OPs2, OPs3), HbA1c, UACR, UA, TG, LDL, HDL and CHO in T2DM with non-smokers.^*^**

|  | fAGEs | OPs1 | OPs2 | OPs3 | HBA1c | UACR | UA | TG | LDL | HDL | CHO |
| --- | --- | --- | --- | --- | --- | --- | --- | --- | --- | --- | --- |
| fAGEs | 1.000 | 0.644 | 0.653 | 0.856 | 0.027 | 0.076 | -0.024 | -0.119 | -0.112 | 0.001 | -0.115 |
| OPs1 | 0.644 | 1.000 | 0.940 | 0.702 | 0.055 | 0.107 | 0.027 | -0.103 | -0.038 | 0.041 | -0.050 |
| OPs2 | 0.653 | 0.940 | 1.000 | 0.697 | -0.011 | 0.208 | 0.023 | -0.094 | 0.020 | 0.030 | 0.008 |
| OPs3 | 0.856 | 0.702 | 0.697 | 1.000 | -0.007 | 0.111 | -0.059 | -0.113 | -0.139 | -0.089 | -0.161 |
| HBA1c | 0.027 | 0.055 | -0.011 | -0.007 | 1.000 | -0.073 | -0.034 | 0.102 | 0.023 | -0.091 | -0.022 |
| UACR | 0.076 | 0.107 | 0.208 | 0.111 | -0.073 | 1.000 | -0.027 | 0.041 | 0.482 | 0.351 | 0.522 |
| UA | -0.024 | 0.027 | 0.023 | -0.059 | -0.034 | -0.027 | 1.000 | 0.146 | -0.122 | -0.227 | -0.128 |
| TG | -0.119 | -0.103 | -0.094 | -0.113 | 0.102 | 0.041 | 0.146 | 1.000 | 0.229 | -0.200 | 0.232 |
| LDL | -0.112 | -0.038 | 0.020 | -0.139 | 0.023 | 0.482 | -0.122 | 0.229 | 1.000 | 0.395 | 0.959 |
| HDL | 0.001 | 0.041 | 0.030 | -0.089 | -0.091 | 0.351 | -0.227 | -0.200 | 0.395 | 1.000 | 0.558 |
| CHO | -0.115 | -0.050 | 0.008 | -0.161 | -0.022 | 0.522 | -0.128 | 0.232 | 0.959 | 0.558 | 1.000 |

^*^ fAGEs, Fluorescent advanced glycation end products; OPs1, dityrosine; OPs2, N'-formylkynurenine; OPs3, kynurenine; HbA1c, hemoglobin A1C; UACR, urinary albumin/creatinine ratio; UA, serum uric acid; TG, triglyceride; LDL, low-density lipoprotein; HDL, high-density lipoprotein, CHO, cholesterol; T2DM, type 2 diabetes mellitus; CIMT, carotid intima media thickness.

**Supplemental table 7 Pearson's correlation coefficient (r) of fAGEs, protein oxidation products (OPs1, OPs2, OPs3), HbA1c, UACR, UA, TG, LDL, HDL and CHO in T2DM with smokers.^*^**

|  | fAGEs | OPs1 | OPs2 | OPs3 | HBA1c | UACR | UA | TG | LDL | HDL | CHO |
| --- | --- | --- | --- | --- | --- | --- | --- | --- | --- | --- | --- |
| fAGEs | 1.000 | 0.870 | 0.877 | 0.906 | 0.305 | 0.375 | 0.108 | -0.089 | 0.061 | 0.036 | 0.019 |
| OPs1 | 0.870 | 1.000 | 0.962 | 0.772 | 0.225 | 0.350 | 0.041 | -0.011 | 0.097 | 0.025 | 0.073 |
| OPs2 | 0.877 | 0.962 | 1.000 | 0.831 | 0.214 | 0.406 | 0.105 | -0.041 | 0.109 | -0.012 | 0.064 |
| OPs3 | 0.906 | 0.772 | 0.831 | 1.000 | 0.246 | 0.420 | 0.091 | -0.179 | 0.008 | 0.019 | -0.048 |
| HBA1c | 0.305 | 0.225 | 0.214 | 0.246 | 1.000 | 0.026 | -0.033 | 0.022 | 0.037 | -0.029 | 0.014 |
| UACR | 0.375 | 0.350 | 0.406 | 0.420 | 0.026 | 1.000 | -0.169 | 0.045 | -0.067 | -0.124 | -0.054 |
| UA | 0.108 | 0.041 | 0.105 | 0.091 | -0.033 | -0.169 | 1.000 | 0.202 | 0.103 | 0.117 | 0.152 |
| TG | -0.089 | -0.011 | -0.041 | -0.179 | 0.022 | 0.045 | 0.202 | 1.000 | 0.374 | -0.029 | 0.589 |
| LDL | 0.061 | 0.097 | 0.109 | 0.008 | 0.037 | -0.066 | 0.103 | 0.374 | 1.000 | -0.037 | 0.940 |
| HDL | 0.036 | 0.025 | -0.012 | 0.019 | -0.030 | -0.124 | 0.117 | -0.029 | -0.037 | 1.000 | 0.170 |
| CHO | 0.019 | 0.073 | 0.064 | -0.048 | 0.014 | -0.054 | 0.152 | 0.589 | 0.940 | 0.170 | 1.000 |

^*^ fAGEs, Fluorescent advanced glycation end products; OPs1, dityrosine; OPs2, N'-formylkynurenine; OPs3, kynurenine; HbA1c, hemoglobin A1C; UACR, urinary albumin/creatinine ratio; UA, serum uric acid; TG, triglyceride; LDL, low-density lipoprotein; HDL, high-density lipoprotein, CHO, cholesterol; T2DM, type 2 diabetes mellitus; CIMT, carotid intima media thickness.
